# Supplementary figures and images for: The effect of yoga and aerobic exercise on children’s physical activity in rural India: a randomized controlled trial
Source: PeerJ. 2025 Jul 8;13:e19604. doi: 10.7717/peerj.19604 (PMC12248224; doi:10.7717/peerj.19604)

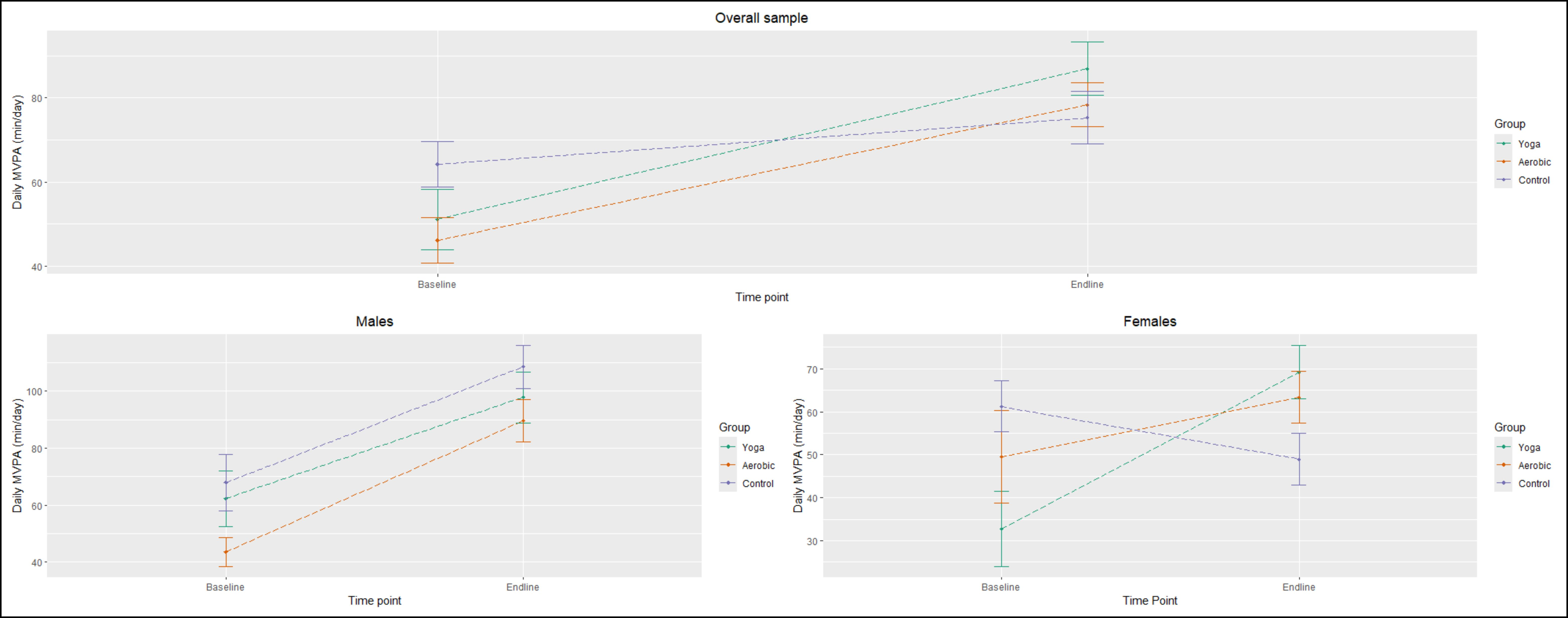

Supplement: Supplemental Information 1 — Yoga intervention group: green; aerobic intervention group: orange; and control group: purple. First panel: overall sample; second panel: male subsample; and third panel: female subsample. [file peerj-13-19604-s001.png]
