# Supplementary material for: The effect of yoga and aerobic exercise on children’s physical activity in rural India: a randomized controlled trial
Source: PeerJ. 2025 Jul 8;13:e19604. doi: 10.7717/peerj.19604 (PMC12248224; doi:10.7717/peerj.19604)
Supplement: Supplemental Information 2 [file peerj-13-19604-s002.docx]

**DETAILS OF THE RESEARCH PROJECT**

**TITLE OF THE PROJECT**: Studies in Inter relations of Physical Activity, Nutrient Intake and Muscle Function in Rural Indian Children

1**. Aim**: To assess muscle function parameters and determinants of muscle function and to evaluate the effects of nutritional supplementation with or without exercise intervention on muscle function in rural Indian school going children.

2. **Objectives:**

1. To establish gender specific paediatric and adult reference data for muscle function parameters assessed using jumping mechanography.
2. To investigate the determinants of muscle mass and function in rural Indian school children aged 6-10y.
3. To evaluate the effect of nutritional supplementation and exercise intervention on muscle function in rural Indian school children.

3. **Lacuna in knowledge on the subjects (briefly):**

The importance of muscle mass, strength, and metabolic function in the performance of exercise, as well as the activities of daily living is very well known. Muscle mass is often used as a surrogate for muscle function; however, functional capacity can be increased without changes in muscle mass. Muscle function includes force (the product of mass and acceleration), and power. Furthermore, Anliker et al, recently described a strong correlation between the maximum force (Fmax) of lower body muscles and bone mineral content (BMC) of the tibia in children and adults and showed that Fmax predicted 84% of the BMC. In another study done in men aged 25-45 years, muscle force and power, were positively correlated with cortical bone area and cortical thickness. Importantly, population studies involving older men and women have shown that the decline in muscle force is signiﬁcantly greater than the decline in muscle mass, indicating that the force generated by muscle contractions is not proportional to muscle mass. **Hence assessment of muscle function is very essential.** Among the techniques used for muscle assessment, measurement of grip force by dynamometry is the most widely used test. This method, however, has limited relevance for the examination of muscle force because it only assesses force at the upper extremity, i.e., at the non-weight-bearing part of the body. Also, dynamometry does not mirror the movement patterns used during daily physical activities. **Hence there is a need for a better, reliable technique for muscle function assessment.** Newer techniques such as the portable ground reaction force plates (GRFP), which can examine dynamic muscle function, seem promising as simple and reliable methods for assessing muscle function. Jumping mechanography GRFP was designed to measure muscle force and power by deriving measurements from an individual’s ground reaction forces. However, one of the limitations of this method is the lack of ethnicity matched pediatric and adult reference data. The available published reference values are mostly from European countries. To enable jumping mechanography to become widely usable in clinical practice as a basic screening method for muscle function assessment, there is a need to examine a larger population. **Also, for the correct interpretation of jumping mechanography measurements, the use of age, gender, height, weight and ethnicity specific reference data is very important.**

Weight-bearing activities during childhood have a positive effect on bone strength and low bone strength in childhood is associated with a higher fracture risk in later life. The fracture risk relates to bone strength, muscle strength and postural balance. In a study, 3×40 min/week exercise intervention over a full school year improved several aspects of muscular ﬁtness of children aged 8 – 10 years, suggesting that well-organised intense physical education classes can contribute positively to develop musculoskeletal health in young children. In another study conducted in 8-12 year old girls and boys to see the effects of jumping exercise on maximum ground reaction force and bone, no significant changes were seen in the intervention and control groups’ maximum ground reaction force. The effects of these programs are limited and the findings are mixed. The mixed findings may be because many traits like sex, maturity, age, weight, height and ethnicity influence muscle strength. Exercise initiation during the prepubertal years proves to be the most beneficial in improving the musculoskeletal system than activity undertaken during puberty. It is important to identify safe and effective exercise or PA programs that can improve musculoskeletal health during growth in Indian children. There is considerable evidence that increased dietary protein combined with progressive resistance training can enhance muscle mass, size, and strength, particularly in young- and middle-aged adults. However, such data is scarce in paediatric population. Also, whether exercise combined with a high protein diet or protein supplementation can promote a greater response than either exercise or protein alone has not been investigated. **It is, thus, worthwhile to study the effects of different types of exercises along with protein and energy supplementation on muscle function parameters assessed using jumping mechanography.**

**1. DETAILED RESEARCH PLAN**

The reference data will be generated separately for the three age groups; 5-18y, 18-50y and 50y onwards considering the muscle status and level of physical activity. A random sample of 40 healthy boys and 40 healthy girls for each age group from 5 – 18 years (total n=960) and for 20-80 years: 40 men and 40 women in each decade (n= 480) would be selected by stratified sampling method. Thus, the total sample size for this study is estimated to be 1440 throughout different age groups. Healthy, physically active subjects within the age range of 6 to 80 years, will be recruited from schools, colleges, offices residing in and around Pune city.

For the 3-arm intervention study, a total of 225 children (75 in each group) aged 6-10 years will be enrolled. Healthy, physically active children, aged 6-10 years, from rural schools in Pune district.

1. Anthropometric measurements namely height, weight will be measured.

2. Blood sample (7-8 ml) will be collected for the assessment of Sr. Calcium, Sr. Phosphorus, Vitamin D, Parathyroid Hormone. (In RCT only)

3. Medical history, history of fractures including age at fracture, site, and will be recorded. Information on habitual physical activity, sunlight exposure and, dietary intake will be collected.

4. Clinical assessment and tanner staging (in children) will be conducted by a trained paediatrician.

5. Body composition and muscle strength will be measured.

**2. STUDY SITES:**

1. Hirabai Cowasji Jehangir Medical Research Institute, Pune.

2. Zilla Parishad schools in Pune district

**Study Design**: 1. Cross sectional study 2. Randomized controlled trial (RCT)

**Time Perspective**: 3 years.

**Inclusion criteria:**

1. Apparently healthy 6 to 80 year old children and adults
2. Apparently healthy children from 6 to 10 years of age.

**Exclusion criteria**:

- For 6-18 years: Height or weight below 3^rd^ percentile or above 97^th^ percentile according to Indian reference charts recommended by the Indian Academy of Pediatrics.
- For 19-80 years: BMI below or above the reference range
- Prolonged periods of immobilization in the past 12 months
- Use of any drug known to affect bone or muscle health
- Subjects who have received Vitamin D supplements previously

**Work Plan and Methods**:

**Time Schedule:**

**Study Period:** 32 months

Participant Enrolment for reference data: 12 months

Participant enrolment for RCT: 2 months

Intervention period: 6 months

Data Entry and Cleaning: 6 Months

Generating and Publishing Reference centiles: 6 Months

Total: 32 Months

**Study Design**:

Cross-sectional study, 6-10 year old, n = 225

Randomized Placebo Controlled Trial, 6 -10 year old, n = 225

Random allocation to 3 groups

Protein + Exercise Module1 intervention n=75

Protein + Exercise Module2 intervention n=75

Only Protein Supplementation n=75

n=75

Intervention – 6 months

Post Intervention Measurements: All pre-intervention measurements will be repeated

Statistical Analysis of Data and Interpretation of Results

Reference Data, 6-80 year old, n = 1440

Urban Population

Rural Population

Measurements: Demographic information, anthropometry, PA recall, Sunlight exposure questionnaire, body composition by BIA, Muscle function tests by Jumping Mechanography

Measurements: Demographic information, clinical examination, biochemistry, anthropometry, PA recall, Sunlight exposure questionnaire, body composition by BIA, diet recall, muscle function tests by Jumping Mechanography (Baseline measurements for RCT)

Studies in muscle function parameters

**Tools for Measurement**:

Using a structured questionnaire and by face-to-face interview, the following information will be collected:

1) Demographic data, socio-economic status data, dietary information, physical activity, medical and fracture history will be collected with the use of a questionnaire.

2) Nutrient intakes will be estimated by C-Diet software.

3) Standard stadiometer and weighing scales will be used for height, weight.

4) Jumping mechanography GRFP will be used to measure lower body muscle strength. Digital Hand Dynamometer will be used to measure upper body muscle strength.

5) The hemoglobin will be measured by finger prick method by Hemocue.

6) Blood sample (7-8 ml) will be collected by a trained paediatric nurse for assessing Sr. Calcium, Sr. Phosphorus, vitamin D3 and PTH.

7) The body composition assessment will be performed by bioelectrical impedance analysis (BIA) technique (TANITA BC 420)

Selection of Subjects: After satisfying inclusion/exclusion criteria, selection will be on a

random basis

Statistical Analysis Plan:

Statistical analysis will be performed using SPSS. Summary statistics will be described as mean

and SD. In case of non-normal variables, median and IQR will be used. The LMS chart maker

program will be used to generate the reference centile curves. Student’s t-test will be used to

examine the significance of difference between the means of body composition and muscle

parameters in the 3 groups. One-way ANOVA will be used to examine the significance of

difference between the means of body composition and muscle parameters in various age

groups.

Correlation coefficient analysis will be performed to study associations between various lifestyle factors and muscle strength parameter. Regression analysis will be carried out to assess the determinants of muscle parameters.

Ethical Issues: Ethical approval will be sought from institutional ethics committee before the

study commences. Written Informed consent and assent will be obtained from participants.
